# Single molecule analysis of *Trypanosoma brucei* DNA replication dynamics

**DOI:** 10.1093/nar/gku1389

**Published:** 2015-02-17

**Authors:** Simone Guedes Calderano, William C. Drosopoulos, Marina Mônaco Quaresma, Catarina A. Marques, Settapong Kosiyatrakul, Richard McCulloch, Carl L. Schildkraut, Maria Carolina Elias

**Affiliations:** 1 Laboratório Especial de Ciclo Celular, Instituto Butantan, São Paulo, SP 05503–900, Brasil; 2 Center of Toxins, Immune Response and Cell Signaling – CeTICS, Instituto Butantan, São Paulo, SP 05503–900, Brasil; 3 Department of Cell Biology, Albert Einstein College of Medicine, Bronx, NY 10461, USA; 4 The Wellcome Trust Centre for Molecular Parasitology, College of Medical, Veterinary and Life Sciences, Institute of Infection, Immunity and Inflammation, University of Glasgow, Glasgow, G128TA, UK

## Abstract

Eukaryotic genome duplication relies on origins of replication, distributed over multiple chromosomes, to initiate DNA replication. A recent genome-wide analysis of *Trypanosoma brucei*, the etiological agent of sleeping sickness, localized its replication origins to the boundaries of multigenic transcription units. To better understand genomic replication in this organism, we examined replication by single molecule analysis of replicated DNA. We determined the average speed of replication forks of procyclic and bloodstream form cells and we found that *T. brucei* DNA replication rate is similar to rates seen in other eukaryotes. We also analyzed the replication dynamics of a central region of chromosome 1 in procyclic forms. We present evidence for replication terminating within the central part of the chromosome and thus emanating from both sides, suggesting a previously unmapped origin toward the 5′ extremity of chromosome 1. Also, termination is not at a fixed location in chromosome 1, but is rather variable. Importantly, we found a replication origin located near an ORC1/CDC6 binding site that is detected after replicative stress induced by hydroxyurea treatment, suggesting it may be a dormant origin activated in response to replicative stress. Collectively, our findings support the existence of more replication origins in *T. brucei* than previously appreciated.

## INTRODUCTION

Chromosomal replication is initiated through the assembly of the pre-replication complex (pre-RC) at DNA sites called origins of replication (1). Unlike in most bacteria, where the bi-directional DNA replication forks emerging from a single replication origin duplicate the entire circular genome, the genomes of eukaryotic cells are replicated from many replication origins distributed along multiple chromosomes. However, the features that define these origins are not well understood in most eukaryotes. Well-defined, conserved sequences that determine the sites of initiation of DNA replication have been identified in *Saccharomyces cerevisiae* (2), where origins are called autonomously replicating sequences. In other eukaryotes consensus sequences have not been described at mapped replication origins, suggesting they can be defined by other parameters, such as nucleosome positioning, histone modifications and 3-D organization of the nucleus (3). The origins distributed throughout eukaryotic genomes display variations in terms of efficiency, including the frequency at which an origin fires within a population of cells and activation time during S-phase. Among this range of efficiency are dormant origins, which are typically silent, but can be activated as backup origins when passive replication from nearby origins is impeded (4–6). Such origins may be crucial, because the replication fork must overcome numerous natural obstacles, including transcription complexes engaged along the same DNA template (7–9).

*Trypanosoma brucei* is the etiological agent of sleeping sickness, which primarily affects human populations in some of the least developed countries of Central Africa. During its life cycle, *T. brucei* alternates between insect and mammalian hosts and the life cycle of this parasite includes forms that are able to proliferate and forms that do not replicate (10). Since infective forms are not able to replicate their DNA, blockage of DNA replication may play a role in *T. brucei* transmission. The 26-megabase genome of *T. brucei* was sequenced in 2005 (11), in conjunction with two other related kinetoplastid parasites, revealing a highly unusual genomic organization, with each chromosome containing directional gene clusters (DGCs) comprising an average of 50 (but sometimes hundreds) of genes (12). Most DGCs are transcribed (probably constitutively) by RNA polymerase II, producing polycistronic transcripts that are processed into single-gene mRNAs through trans-splicing (13,14). Nuclear DNA replication in *T. brucei* and its relatives has only begun to be characterized. Our group identified a component of the pre-RC, the initiator machinery present at replication origins, of *T. brucei*. By its homology with the two pre-RC components Orc1 and Cdc6, we designated it ORC1/CDC6. ORC1/CDC6 is able to complement yeast Cdc6 and the lack of ORC1/CDC6 results in arrested DNA replication in *T. brucei* (15,16). To date, ORC1/CDC6 is the only validated replication initiation factor in *T. brucei*, though several additional factors with which it interacts have been described (17–19). Recent global analysis mapped early replicating origins in *T. brucei*, indicating a remarkably small number of active origins in this organism, each of which localized to the boundaries of the DGCs (20). Moreover, the mapped origins were not found at all DGCs and they were found at only a fraction of the mapped binding sites for ORC1/CDC6. Thus, questions remain about replication dynamics in *T. brucei*. For instance, what distinguishes the DGC boundaries and ORC1/CDC6 sites that are used as origins from those that are not? Furthermore, genomic plasticity provides a mechanism for switching cell surface proteins to escape the mammalian immune system involving high rates of recombination (21) and it is unknown if and how DNA replication might influence this process. For these reasons, and others, it is important to thoroughly characterize DNA replication in *T. brucei* and related organisms (19).

In the present study, we applied single molecule analysis of replicated DNA (SMARD) to characterize the dynamics of *T. brucei* DNA replication through S phase. Analysis of 150 kb fragments from throughout the *T. brucei* genome revealed replication rates in procyclic form cells (replicative form living in the fly vector midgut) and bloodstream form cells (replicative forms living in the mammalian blood) to be 3.7 ± 0.1 and 4.4 ± 0.1 kb/min, respectively. To further analyze the dynamics of DNA replication in this organism, we examined a specific 347 kb region in the middle of chromosome 1. We found that this region is a zone of replication termination in *T. brucei* procyclic forms, with forks arriving from 3′ (most likely from a previously mapped centromeric origin) and from 5′. As no origins have been mapped 5′ of this region of chromosome 1, these data imply that there are more origins in the *T. brucei* genome than initially described (20). Moreover, termination points are not at a fixed position in chromosome 1, suggesting variation in rate of origin firing or fork progression. Finally, we provide evidence of a dormant origin in this region, which was only detected after hydroxyurea (HU) treatment, suggesting it may activated primarily in response to replicative stress.

## MATERIALS AND METHODS

### Cell culture and synchronization

The procyclic form of *T. brucei* TREU 927 was grown in SDM-79 medium supplemented with 10% fetal serum bovine at 28°C. The bloodstream form of *T. brucei* 2T1 was grown in HMI-9 medium supplemented with 10% fetal serum bovine and 10% serum plus (Sigma) at 37°C. For procyclic synchronization (22), exponential cultures containing 5 × 10^6^ cell/ml were treated with 0.2 mM of HU for 12 h.

### Flow cytometry analysis

A total of 10^6^ cells were washed with phosphate-buffered saline (PBS) and fixed with ice-cold 70% ethanol in PBS at 4°C overnight. Subsequently, the cells were washed once with PBS and resuspended in PBS containing 10 μg/ml of propidium iodide and 100 μg/ml of RNAse A. After 45 min of incubation at 37°C, the cells were analyzed using BD FACSCalibur equipment (Voltage and AmpGain were respectively: FSC E00, 6.23; SSC 334, 3.12 and the AmpGain for FL2-A was 4.26 and for FL2-W was 5.57. Mode of all parameters was linear).

### SMARD

SMARD was performed essentially as described previously (23). Exponential cultures of procyclic (5 × 10^6^/ml) or bloodstream (10^6^/ml) *T. brucei* were pulse-labeled with 100 μM of IdU (Sigma) for 40 min. The cells were centrifuged for 10 min at 10,000 revolutions per minute, and cell pellets were resuspended and incubated with 100 μM CldU (Sigma) in culture medium for 40 min at 28°C. Labeled cells were embedded in 0.5% low melting agarose (InCert; FMC) at 5 × 10^7^ cells per 100 μl agarose cell plug and lysed overnight at 50°C in 1% n-lauroylsarcosine, and 0.5 M ethylenediaminetetraacetic acid (EDTA), pH 8, containing 200 μg/ml proteinase K. The plugs were then washed with TE, pH 8, treated with 200 μM phenylmethanesulfonyl fluoride then washed with TE, pH 8. Subsequently, the plugs were equilibrated in restriction enzyme digestion buffer, then *Asc* I (30 U) was added and the plugs incubated overnight at 37°C. The plugs were then cast into a 0.8% low-melting agarose gel (SeaKem GTG agarose;Lonza) and the DNA separated by pulse field gel electrophoresis (PFGE) using a CHEF Mapper XA (BioRad), see legends of figures for parameters of PFGE. A portion of the gel containing the digested DNA was transferred onto a nylon membrane while the remaining portion was saved. To identify a specific 347 kb fragment from *T. brucei* chromosome 1, a probe was generated by polymerase chain reaction (PCR) (forward ACA GCA GCG AAA GGA TAG GA; reverse GCA AAT GAT GCC AAC AAC AC and genomic DNA as template) and labeled using the Amersham AlkPhos Direct Labeling Kit (GE Life Science). Southern blotting was performed to locate the position of the DNA segments of interest using the chromosome 1 PCR probe. The corresponding gel region (from the previously saved gel) was excised, melted and digested with agarase (GELase;Epicentre). The resulting DNA in solution was stretched on microscope slides coated with 3-aminopropyltriethoxysilane (Sigma). The stretched DNA was denatured for 12 min in alkali buffer (70% ethanol, 0.1% β-mercaptoethanol and 0.1 M NaOH) then fixed for 5 min in alkali buffer containing 0.5% glutaraldehyde, to prevent reannealing of the denatured DNA. The slides were subsequently hybridized overnight with biotinylated 10 kb fluorescence *in situ* hybridization (FISH) probes prepared by PCR (Probe 2: forward GAG GCT CAG TGC TTG CTG AAA CC and reverse CAG GGC CGC CGT CCT TTG TAC; Probe Middle (M): forward GGA ACA TGC AGC ACG GGG CA and reverse GTA ACT GCG GCT GTT GGC GC and Probe 3: forward GCT TTT TGG GGG AAA TGG TTC GC and reverse CCT GGA CTA GGA GCG TCT GG) using an Expand Long Template PCR System (Roche) followed by biotin nick translation (Biotin Nick Translation Mix; Roche). The hybridized slides were blocked for 30 min at room temperature with PBS/1% bovine serum albumin. FISH probes were detected by incubating with an Alexa Fluor 350–conjugated NeutrAvidin antibody (Invitrogen) followed by two rounds of incubation first with a biotinylated anti-avidin antibody (Vector) and then the Alexa Fluor 350-conjugated NeutrAvidin antibody. The two incorporated, halogenated nucleosides were visualized by indirect immunostaining, during the second round of FISH detection using a mouse anti-IdU monoclonal antibody (Becton Dickinson) and a rat anti-CldU monoclonal antibody (Accurate) followed by Alexa Fluor 568-conjugated goat anti-mouse (Molecular Probes) and Alexa Fluor 488-conjugated goat anti-rat antibodies. Alternatively, for slides that were not hybridized with FISH probes (non-specific genomic fragments), anti-IdU immunostaining was performed (as above) followed by immunostaining with an anti-DNA monoclonal antibody (24) followed by anti-mouse immunoglobulin (Alexa fluor 350 goat anti-mouse IgG-Molecular Probes). After FISH and immunostaining the slides were mounted using ProLong Gold antifade (Molecular Probes), and images were acquired using a XM10 camera coupled to a 2.5× magnification lens and a Nikon E600 microscope containing a Nikon 100×/1.3 Plan-apochromatic objective. ­Filters used for image acquisition were U-MWU2, U-MWiBA-3 and U-MWG2 (Olympus/Chroma). Software used was CellF v3.4 (Olympus). Image acquisition was made at 700 ms exposure for green and red filters and 300 ms for blue filter. Image resolution was 1376 × 1032 × 24 pixel.

### Real-time PCR

For the real-time PCR analysis after synchronization, genomic DNA was extracted from procyclic *T. brucei* at the middle and final stages of S phase after release from HU using DNAzol (Invitrogen). Segments of 80 bp were amplified from different regions of chromosome 1 using 50 ng of genomic DNA and 50 nM of each primer with a SYBR Green mix (Applied Biosystems) in a final volume of 20 μl. The following primers were used: ∼397 kb region forward GAA CAA ACG CAT TGG AGG TG and reverse GCA CTT GTT GTC TCC CAA AC; ∼440 kb region forward GTT CCA TGA CTG AGG AGC AG and reverse GTC TCA ACT GGA GGT CGA AG; ∼505 kb region forward CTA CCG ACC GAA AGG AAC TG and reverse CGC TTC AAT CCG AAG CAA AG; ∼550 kb region forward TGG ATG TTC CAC CGC TTT CA and reverse TGT TCT TCA GAT CCT GCG GT; ∼665 kb region forward GGA ATT GGC CCA CAA AAT GG and reverse CAA CAT CAC CGA CTA CCT GG; and ∼798 kb region forward CAA CCG TGA TTC TCT CAG TCA G and reverse CCA CAA AAA TGG TGC CAC AG. The reactions were performed using Step One Plus (Applied Biosystems). The Ct values and primer efficiencies were obtained using the LinRegPCR (2012.x) program. The quantification was obtained using the Pfaffl method (25), in which a sequence from kinetoplast DNA was used as the reference (forward GGA GAT TCT TGG GGA GAG GC and reverse GCA ATT CCC AAT TCC ATT TCC C), and DNA from cells at G2 phase was used as a control. For marker frequency analysis (MFA) by qPCR without HU synchronization, genomic DNA was extracted from procyclic *T. brucei* cells sorted by fluorescent-activated cell sorting (FACS) into early S, late S and G2 phases, as described (20). Briefly, ∼1 × 10^9^ cells were collected from an exponentially growing procyclic cell form culture (between 5 × 10^6^ and 1 × 10^7^ cells/ml), and centrifuged for 10 min at 1620 × *g*. The pellet was then washed in 10 ml of 1× PBS supplemented with 5 mM EDTA (Gibco), and centrifuged for 10 min at 1620 × *g*. Next, the cells were re-suspended in 12 ml of 1× PBS supplemented with 5 mM of EDTA, to which 28 ml of 100% ice cold-Methanol was added, in a drop-wise fashion while vortexing gently, so that the final fixing solution was of 70% (v/v) Methanol, and the cell concentration of 2.5 × 10^7^ cells/ml. The tube was wrapped in aluminum foil paper and kept at 4°C from overnight up to 3 weeks. For every FACS sorting run, four FACS tubes (Becton Dickinson) were prepared, each starting with 4 ml of fixed cells (1 × 10^8^ cells). The cells were collected and centrifuged for 10 min at 1000 × *g*, at 4°C, washed in 1 ml of 1× PBS supplemented with 5 mM of EDTA and centrifuged again for 10 min at 1000 × *g*, at 4°C. The pellet was then re-suspended in 4 ml of 1× PBS supplemented with 5 mM of EDTA, 10 μg/ml of propidium iodide (Sigma Aldrich) and 10 μg/ml of RNase A (Sigma Aldrich), and incubated for 45 min at 37°C, in the dark. The cells were then transferred to a FACS tube through a cell strainer cap (Becton Dickinson), and sorted into G1, early S, late S and G2 phases using a BD FACSAria I Cell Sorter (Becton Dickinson). An average of 1 × 10^7^ cells were recovered for the G1 subset, 1 × 10^6^ cells for both early and late S phases and 3 × 10^6^ cells for G2 phase, per FACS sorting session (three sessions were done in total for each *T. brucei* strains). The sorted cells were collected at 4°C into new FACS tubes containing 200 μl of lysis buffer (1 M NaCl, 10 mM EDTA, 50 mM Tris-HCL pH 8.0, 0.5% sodium dodecyl sulphate, 0.4 mg/ml Proteinase K and 0.8 μg/ml of Glycogen) (26). The collected cells were then incubated for 2 h at 55°C, and the lysate was stored at −20°C until gDNA extraction. gDNA was extracted using the Blood and Tissue DNA extraction kit, from Qiagen, following the steps downstream of the lysis instructions. The purified gDNA was then eluted in buffer AE and its concentration determined using a Qubit 2.0 (Invitrogen). In total, three independent qPCR runs were performed with the same samples, and in each individual run triplicates for each pair of primers described above were performed. All experiments were performed in MicroAmp R Optical 96-well Reaction Plates (Life Technologies). Due to the low amounts of sample, SYBR Select Master mix (Life Technologies) was chosen, and used with 400 nM of primers and 0.01 ng of genomic DNA, to a total of 20 μl per reaction. The experiments were performed in a 7500 Real Time PCR system (Applied Biosystems), according to the SYBR Select Master Mix manufacturer's PCR cycling instructions: 50°C for 2 min and 95°C for 2 min, followed by 40 cycles of 95°C for 15 s, 59°C for 15 s and 72°C for 1 min. In order to confirm the specificity of the reaction, a final dissociation step was included in the program. The resulting fluorescence intensity data (collected at the end of the extension step, 72°C for 1 min) was then analyzed by relative quantification using the ΔΔ*C_t_* method (7500 software version 2.3, Applied Biosystems), with the primers targeting the kinetoplast DNA (kDNA) being used as the endogenous control and the G2 phase sample as the calibrator. For representation, ΔΔ*C_t_* data from Early S phase was plotted against the G2 phase in the form of points (representing the mean of the three qPCR runs) and connecting lines; error bars represent the standard error of the mean. Graphs were generated using GraphPad Prism version 5.03.

## RESULTS

### DNA replication rate in procyclic and bloodstream life cycle forms

**Figure 1.**
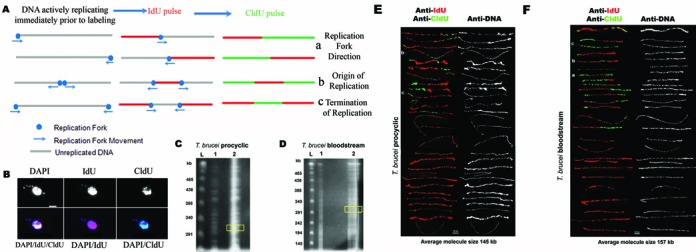
The *T. brucei* replication rate is similar to rates of other eukaryotes. (**A**) Schematic representation of replication analysis by sequential pulse labeling. Actively replicating DNA in exponentially growing cells is labeled by performing consecutive pulses of growth in the presence of thymidine analogs IdU or CldU. The incorporated analogs are identified by immunostaining, appearing as red (IdU) or green (CldU) signals. The temporal order of replication is established by the sequence of labeling (red followed by green). The resulting distinct labeling patterns in individual molecules identify replication forks active during the transition from IdU (red) to CldU pulsing (green) (a), as well as replication initiation (b) and termination events (c). (**B**) IdU and CldU detection on *T. brucei* procyclic form. IdU in red, CldU in green and DAPI in blue. N is the nucleus and k is the kinetoplast (the DNA from the single mitochondria). (**C** and **D**) Nucleoside-labeled genomic DNA (gDNA) from two different forms of *T. brucei* were digested with the restriction enzyme *Asc* I and separated by PFGE using a CHEF Mapper (BioRad), under the following conditions: two-state program, 120° included angle, 6 V/cm, 18 s initial pulse, 33 s final pulse, 30 h running time, linear ramping. L-Lambda Ladder PFG marker (NEB); 1-digested DNA from one agarose plug and 2-digested DNA from four agarose plugs, where a region was cut out for DNA isolation. The yellow boxes indicate the region from where the DNA was recovered. (**E** and **F**) The DNA molecules were stretched on silanized glass slides and nucleoside labeling detected with antibodies against IdU (red), CldU (green) and single-stranded DNA (white). (**E**) Alignment of DNA molecules from procyclic form of 145 kb average length size. (**F**) Alignment of DNA molecules from bloodstream form of 157 kb average size length. These molecules were used to estimate the average replication rate.

To examine DNA replication in *T. brucei*, we used SMARD. In SMARD, actively replicating DNA is sequentially labeled with two distinguishable thymidine analogs, which are detected by immunostaining as distinct (red or green) signals in the labeled DNA (Figure 1A). The sequence of labeling—red followed by green—establishes the temporal order of replication. Therefore, the direction of replication fork progression is visually detected as a transition from red to green labeling (Figure 1A-a). Since replication forks extend bidirectionally from origins, a red region flanked by green regions indicates a replication origin (Figure 1A-b). DNA replication ends when two forks converge, and therefore a green region flanked by red regions indicates a termination event (Figure 1A-c). Thus, SMARD allows the determination of the position, direction and density of the replication forks in a steady-state population of replicating molecules. Moreover, the average speed of the DNA replication forks can be calculated from the observed proportions of differently labeled molecules of the same size (23) (see below).

We initially investigated the rate of DNA replication, comparing *T. brucei* procyclic and bloodstream life cycle forms. Asynchronous populations of cells were sequentially labeled with 5′-iodo-2′-deoxyuridine (IdU) and 5′-chloro-2′-deoxyuridine (CldU) (Figure 1B). Subsequently, the genomic DNA was digested in agarose plugs with *Asc* I, and separated by PFGE, and 300 kb fragments were recovered from the agarose gel (Figure 1C and D). These fragments were stretched on microscope slides and the incorporation of the nucleoside analogs was detected by immunofluorescence. In addition, the molecules were incubated with an anti-DNA antibody for visualization of the entire analyzed molecule, including unlabeled regions. The molecules from procyclic *T. brucei* forms averaged 145 kb in size (Figure 1E), while the bloodstream forms averaged 157 kb in size (Figure 1F). The size of each molecule was calculated using the proportion of 61.54 pixels/kb, obtained from the distance between two defined sequences (identified through hybridization [Figure 3]). The observed lengths obtained indicated that molecules were broken after extraction, as 300 kb molecules were extracted from the agarose gel. Therefore, molecules of similar size were randomly selected, shown in Figure 1E and F, to allow for accurate fork rate measurement. We determined the duplication time (Td) of the analyzed molecules, based on the pulse time of each nucleoside analog (Tp), the number of molecules fully labeled in red (NR) and the number of molecules labeled in red and green (NRG), according to the formula:

\begin{equation*} {\rm Td} = {\rm (TpxNRG)}/{\rm (NRG} + {\rm NR)}(23) \end{equation*}

**Figure 2.**
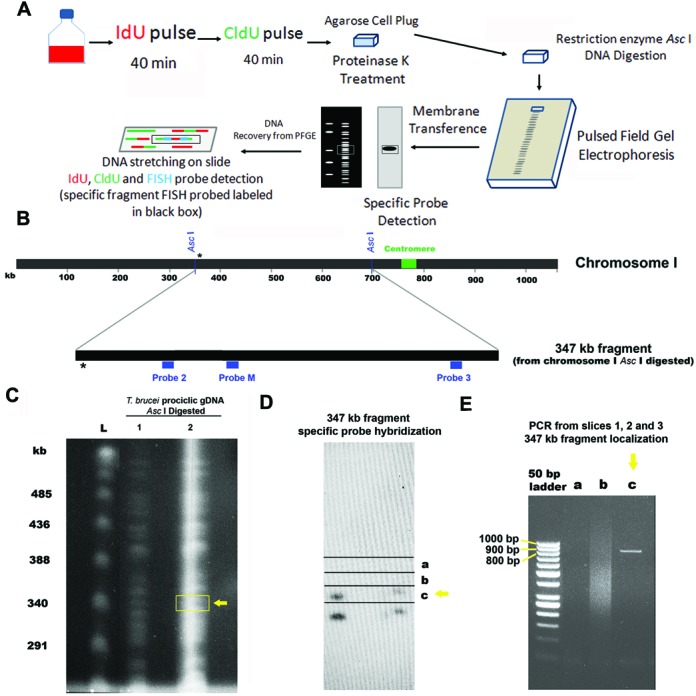
Identification and isolation of a 347 kb fragment from chromosome 1 of *T. brucei* procyclic form. **A**. Schematic representation of SMARD methodology. Consecutive 40 min pulses of thymidine analogs IdU and CldU are performed on an exponential growing cell culture. The cells were cast into agarose plugs then treated with proteinase K. The DNA remaining in the agarose plug was digested with the restriction enzyme *Asc* I and subjected to PFGE. Part of the pulse field gel was transferred to nylon membrane, which was hybridized with a probe specific for the target fragment. A gel slice containing the DNA of interest was excised from the gel and after the agarose was melted and digested the DNA was stretched on silanized slides. The slides were hybridized with specific FISH probes to identify the target fragment. IdU, CldU and FISH probes were detected by indirect immunofluorescence using specific antibodies. Red, green and blue regions on the DNA molecule are IdU, CldU and FISH probe signals, respectively. (**B**) Schematic map of *T. brucei* chromosome 1 (∼1.06 Mb in length) and the 347 kb fragment obtained by *Asc* I digestion of chromosome 1. The centromere (from ∼760 to 790 kb) is marked by a green box. The blue boxes under the 347 kb fragment represent the probes used to identify this fragment on slides prepared for SMARD. The asterisk shows the region targeted by the specific probe used to identify the 347 kb fragment on Southern blots. (**C**) The same pulsed field gel from Figure 1C was transferred to a nylon membrane and (**D**) hybridized with a specific probe to identify the 347 kb fragment. The gel was cut into slices (a, b and c) in order to enrich for the 347 kb fragment. (**E**) PCR was performed on the DNA from the slices using a specific pair of primers to identify the 347 kb fragment. (**C** and **E**) The yellow arrow indicates the position where the 347 kb fragment was detected.

**Figure 3.**
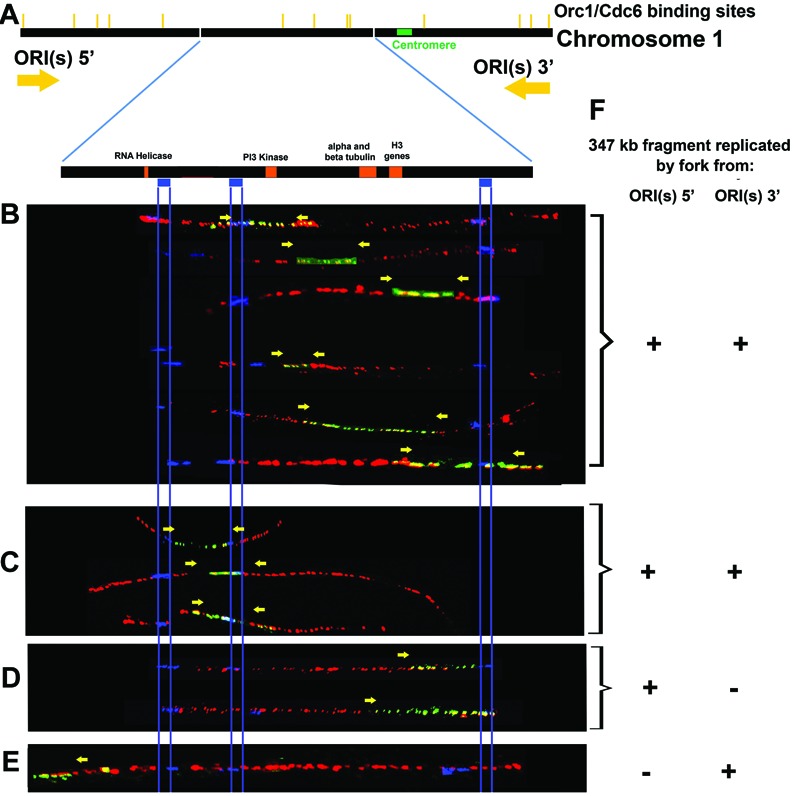
The 347-kb fragment from *T. brucei* chromosome 1 can be replicated by forks from different origins. (**A**) The 347-kb fragment schematic representation where: the yellow bars indicate Orc1/Cdc6 binding sites; the orange boxes indicate some of the genes within this sequence; the blue boxes indicate locations of the FISH probes used to identify the 347-kb fragment and determine its 5′-3′ orientation; the yellow arrows above show the fork direction of different origins of replication. From (**B**) to (**E**): Labeled molecules, with the yellow arrows at points of transition between IdU (red) and CldU (green) incorporation, indicating the fork direction; the blue lines show probe boundaries and are used to align the molecules. (**F**) Indication of which origin (s) of replication (ORI(s) 5′ and ORI(s) 3′) were involved in the replication of the particular molecule.

As indicated by the equation, duplication times are calculated from the proportions of one- and two-colored molecules. Molecules that require longer times to replicate are less likely to complete replication during a single labeling pulse, and thus display a higher ratio of two-color to one-color molecules compared to faster-replicating molecules. The calculated duplication time was 22.22 min for the procyclic form and 23.08 min for the bloodstream form cells. We then calculated fork rates using the formula:

\begin{equation*} \begin{array}{*{20}l} {{\rm Average}\;{\rm Fork}\;{\rm Rate}\;({\rm kb}/{\rm min}) = } \\ {({\rm Length}\;{\rm of}\;{\rm segment}\;({\rm kb})/{\rm Td}({\rm min}))/} \\ {{\rm Average}\;{\rm number}\;{\rm of}\;{\rm replication}\;{\rm forks}\;{\rm for}\;{\rm the}\;{\rm segment}\;(23).} \\ \end{array} \end{equation*}

Based on the molecules in Figure 1, for the procyclic form DNA there were 27 forks in the 15 RG (red green labeled) molecules, giving an average of 1.8 (=27/15) per molecule. For the bloodstream form, there were 23 forks in the 15 RG molecules, giving an average of 1.53 (=23/15) per molecule. Thus, the fork rates were (145/22.22)/1.8 = 3.6 kb/min (average) for the procyclic form cells and (157/23.08)/1.53 = 4.4 kb/min (average) for the bloodstream form cells. We further calculated DNA replication rates from two additional experiments and averaged the rates from all three experiments (Table 1) to obtain a procyclic DNA replication rate of 3.7 ± 0.1 kb/min and bloodstream DNA replication rate of 4.4 ± 0.1 kb/min. These results suggest that *T. brucei* DNA replication rate is similar to the rates seen in other eukaryotes.

**Table 1.**
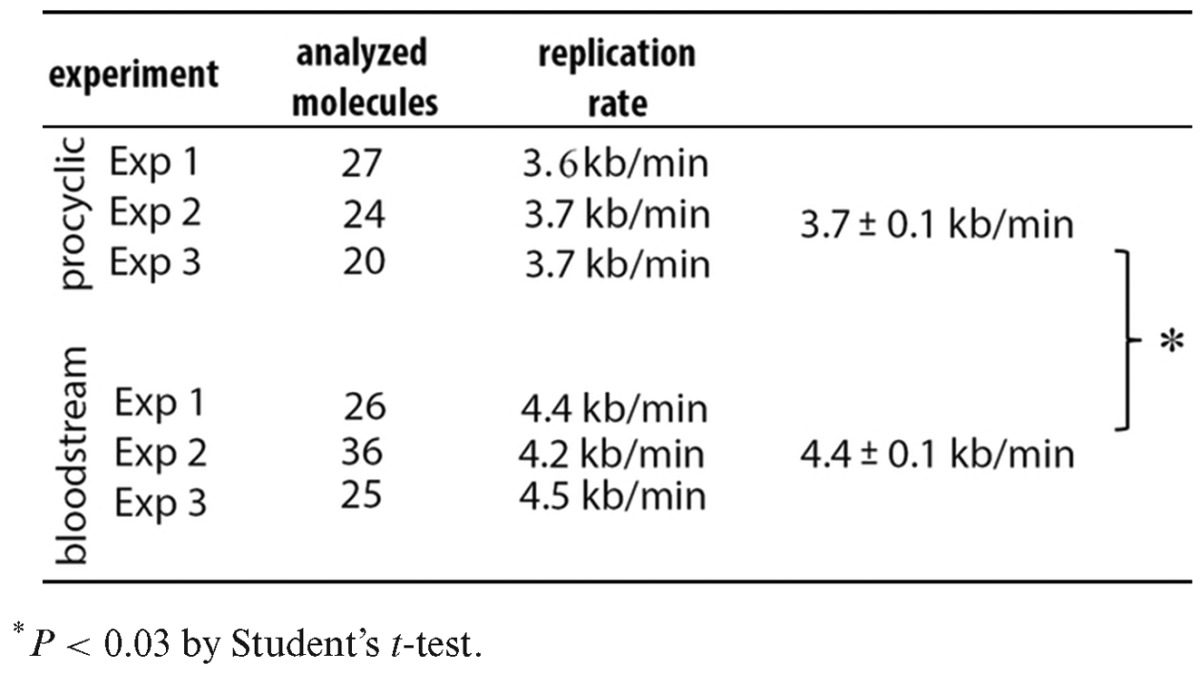
DNA replication rates obtained from three different experiments from procyclic and bloodstream *T. brucei* forms

### DNA replication profile for a 347-kb fragment in the middle of chromosome 1

To further analyze *T. brucei* replication, we selected a specific region in chromosome 1, the smallest of the 11 diploid megabase-sized chromosomes of the *T. brucei* genome, to examine in greater detail. Previous high-throughput analysis of *T. brucei* chromosome 1 identified replication origins close to the single centromere, i.e. around 0.7 and 0.8 Mb from the 5′ end (20). This indicated that a replication fork emerging from the centromeric region at 3.7 kb/min would take ∼200 min to replicate the entire chromosome. Since the *T. brucei* procyclic S phase is only 90 min long (27), this implied that other, unmapped replication origins would be required to fully replicate chromosome 1 within a normal S phase. Therefore, we applied SMARD to examine replication in a region within the middle of chromosome 1, a region sufficiently distant from the centromeric origins to detect replication forks from other origins. Digestion of chromosome 1 with *Asc* I is predicted to generate a 347 kb fragment (from nucleotides (nt) 351,262 to nt 697,918) from the middle of chromosome 1, as shown in Figure 2B. Thus, we performed SMARD analysis on this fragment. As summarized in Figure 2A asynchronous, exponentially growing procyclic form cells were sequentially labeled with IdU and CldU and embedded into agarose plugs. These plugs were proteinase K treated and subsequently the genomic DNA was digested with *Asc* I, subjected to PFGE (Figure 2C), and a portion transferred to a nylon membrane. The membrane was hybridized with a 900 bp probe that binds a sequence present in the 347 kb fragment (nt 360,230 to 361,126; indicated by an asterisk in Figure 2B). Two bands were identified (Figure 2D), most likely corresponding to fragments from the two homologues of chromosome 1 (28), though we cannot say what feature(s) caused the size variation. Because the expected *Asc* I fragment size was 347 kb, the DNA corresponding to the band closest to 347 kb (upper band, Figure 2D) was excised from the gel for further analysis. To confirm that the correct DNA was extracted, the DNA from three different slices (Figure 2D) was recovered and used as templates in PCR reactions containing primers to amplify the specific 900 bp fragment present in the 347 kb fragment (Figure 2E). The DNA recovered from the slice showing a positive PCR signal (slice c; yellow arrow) was used for analysis by SMARD.

The DNA was stretched onto slides and incubated with fluorescent antibodies to detect the halogenated nucleotides. Because the nucleoside analog incorporated during the first pulse (IdU) is labeled in red and the nucleoside incorporated during the second pulse (CldU) is labeled in green, a transition from red to green indicates the direction of replication fork movement. Moreover, as explained above (Figure 1A), two green regions flanking a red region indicates a replication origin, while a green region in the middle of two red regions indicates a termination site. The DNA was also hybridized with specific FISH probes to identify the 347 kb fragment from chromosome 1 among all molecules present on the slide. These three probes were asymmetrically located along the molecule (blue bars in Figures 2B and 3A), allowing for orientation of the visualized fragment. Figure 3B–E shows the molecules obtained. As shown in Figure 3B and C, the most frequent pattern of fluorescence we detected was consistent with replication termination (indicated between the yellow arrows) within the chromosome 1 region. Most of the replication termination events (6/9) occurred in the ‘downstream’ (right) half the region we analyzed (Figure 3B), but some (3/9) were in the ‘upstream’ (left) half (Figure 3C). In a smaller number of molecules, termination was not seen; instead, the patterns indicated replication forks that had traversed the region in a 5′-3′ (Figure 3D; 2 molecules) or 3′-5′ direction (Figure 3E; 1 molecule). Taken together, these data suggest that replication of the central 347 kb region of *T. brucei* chromosome 1 cannot solely be explained by initiation from the centromere-proximal origins mapped previously. Instead, some replication must come from an unmapped origin (or origins) 5′ of the region. Furthermore, because the termination sites occur in a zone rather than at a specific site, either the relative efficiency/frequency of initiation of the 5′ and 3′ origins must vary, or the rate of fork progression is variable. A summary of predicted origin usage is shown in Figure 3F.

In addition, we also identified two molecules potentially containing a replication origin within the 347-kb fragment (Supplementary Figure S1). This origin is located between the first two probes and, unusually, does not colocalize with a mapped ORC1/CDC6 site or boundary of a DGC (data not shown). Nonetheless, it may suggest that the 347-kb fragment can also be replicated by additional origins that have yet to be described, though further analysis will be required to confirm this finding.

### DNA replication emerging from the 5′ region of chromosome 1

**Figure 4.**
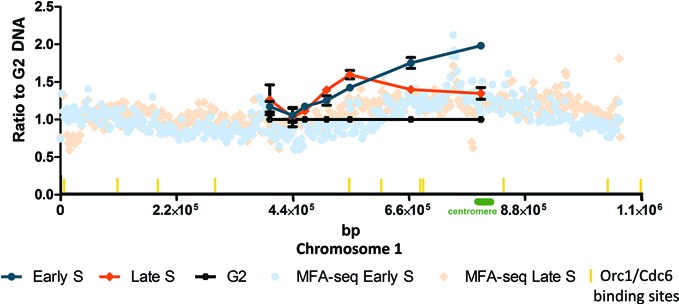
Chromosome 1 replication in unperturbed cells. *T. brucei* replication was mapped by MFA using quantitative PCR, and compared to MFAseq. The line graph shows qPCR at a number of loci within a central 347 kb region of chromosome 1: at each locus the relative quantity of early S phase (blue), late S phase (orange) and G2 phase (black) DNA is shown, in all cases normalized to qPCR of a region of kinetoplast DNA (not shown); G2 values at each locus are set at 1, and the S phase samples shown as a proportion of that value (error bars indicate standard deviation from three experimental repeats). Locations of the qPCR loci within the chromosome (bp) are shown (points in the graph from 5′ to 3′ indicate amplified regions from chromosome 1: nt 397 255–397 535, nt 440 426–440 525, nt 463 829–463 933, nt 504 971–505 071, nt 549 145–549 244, nt 664 782–664 876 and nt 797 547–797 644). MFAseq mapping is shown as dots across the whole chromosome: the ratio of the read-depth between the early (light blue) or late S phase (light orange) and G2 samples is shown, where each dot represents 2500 bp.

Previously, it was shown that there are many ORC1/CDC6 binding sites (potential initiation sites) throughout *T. brucei* chromosome 1 (as seen in all other chromosomes), but MFAseq predicted DNA replication initiation only around the centromere region (20). To compare the above SMARD analysis of the central 347 kb region with the MFAseq, we used qPCR to quantify the amount of DNA in early S and late S replicating procyclic form cells relative to non-replicating G2 cells. To do this, we examined a number of loci across the SMARD-mapped region, with the expectation that replicated loci should display increased DNA abundance in S phase cells relative to late-replicating loci (Figure 4). In this analysis the values at each locus are represented as S/G2 ratios. We used G2 cells to normalize the values because all loci in the chromosome will be replicated in the this cell-cycle phase; indeed, this allows us to directly compare the qPCR data across the 347-kb region with the MFAseq mapping, which covers the whole chromosome (Figure 4) (20). In contrast to previous analysis (20), and to make the data comparable with synchronization experiments (see below), DNA quantity in all the samples was calibrated using qPCR of a kinetoplast DNA (kDNA) sequence. In the S phase samples, there was increasing enrichment relative to G2 as the loci got nearer to the centromere, confirming the MFAseq prediction that replication predominantly initiates around that chromosome feature. In contrast, there was no such S/G2 enrichment at around 0.44 Mb, suggesting that the sequences around here are late-replicating in the region, consistent with the SMARD prediction of termination within the 347-kb region. However, the qPCR displayed only limited evidence for S/G2 enrichment toward the left of the 347-kb region, even in the late S samples. Though this appears inconsistent with the SMARD prediction of replication forks entering the 347-kb region from 5′, it may indicate that replication initiation to the left of the SMARD region is either less efficient than centromere origin-directed initiation, or that initiation occurs at less discrete sites; in both cases, the population-based MFAseq or qPCR approach (used here) would be less accurate than SMARD analysis of individual molecules. Indeed, multiple sites of 5′ initiation may explain the variability in termination location within the 347-kb region.

### A dormant origin is activated in response to DNA replicative stress

**Figure 5.**
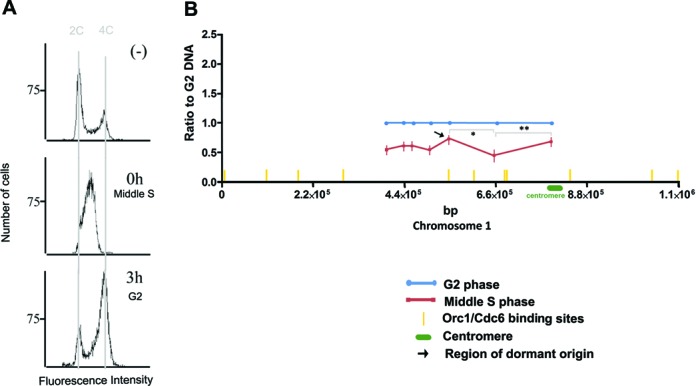
Chromosome 1 replication under replicative stress. *T. brucei* procyclic cells were treated with HU or maintained in culture as control (−). After 12 h of treatment, cells were washed (0 h) and maintained in culture for 3 h. (**A**) Cells were then stained with propidium iodide and analyzed by FACS. (**B**) Additionally, DNA from synchronized cells was extracted and quantified by real time PCR (normalized by regions from kinetoplast DNA) using primers to amplify the regions of chromosome 1 (points in the graph from 5′ to 3′ indicate amplified regions from chromosome 1: nt 397 255–397 535, nt 440 426–440 525, nt 463 829–463 933, nt 504 971–505 071, nt 549 145–549 244, nt 664 782–664 876 and nt 797 547–797 644). *P*-values from Student's *t*-test are *P < 0.03 and **P < 0.02.

Across chromosome 1 there are at least 10 predicted ORC1/CDC6 binding sites, of which 4 are found within the 347-kb SMARD region. To ask if any of these 4 ORC1/CDC6 binding sites within the 347-kb segment have the capacity to act as origins, beyond those around the centromere, we exposed the cells to HU, a well-characterized replicative stress that has been shown to induce dormant origins to activate when forks are stalled for prolonged periods in other eukaryotes (29). Procyclic form *T. brucei* cells were treated with HU for 12 h at a concentration that has been shown to slow S phase progression and provide some cell-cycle synchronization (22). Cell-cycle profiles (Figure 5A) showed that after 12 h of HU treatment, most cells were in the middle of S-phase, and DNA was extracted from this population. In addition, HU was removed and cells allowed to grow for a further 3 h, when most cells were in the G2 stage of the cell cycle (Figure 5A). Again, DNA was extracted from these samples. The amount of DNA in the HU-treated S phase cells relative to the G2 cells was then evaluated at the different loci in the 347-kb region by qPCR, using the same primers as described above for unsynchronized cells. In this HU analysis, as in the unsynchronized cells above, we used primers specific for kinetoplast DNA as an internal control for DNA abundance. Kinetoplast S-phase is completed in the middle of the nuclear S-phase in *T. brucei* (27), with kDNA segregation completed before the nucleus. In the HU conditions used here, though nuclear replication is slowed, kDNA replication is not, and after 12 h of treatment the kDNA network has duplicated but not divided (22). In the 3 h after removal of HU, the kDNA is likely to divide while nuclear DNA synthesis restarts. Thus, in this experiment (unlike the unsynchronized analysis above; Figure 4) the amount of kDNA in middle S (nuclear arrested) cells is equivalent to the amount of kDNA in G2 cells (3 h after HU release). As a result, when we use the kDNA qPCR to normalize the amount of DNA in each PCR reaction (Figure 5B), the level of S is below G2 (and distinct from the unsynchronized analysis; Figure 4). Nonetheless, the variation in S/G2 ratios at each locus provides the same measure of replication across the 347-kb region and reveals the effect of HU treatment. In unsynchronized cells (Figure 4) the highest S/G2 ratio was seen at the rightmost locus, with decreasing S/G2 ratios until around ∼0.44 Mb. In contrast, after HU treatment, the same slope is curtailed, with the lowest S/G2 ratio at ∼0.66 Mb, consistent with replication from the centromeric origin(s) having been stalled. More strikingly, a pronounced S/G2 peak is seen after HU treatment at ∼0.55 Mb, which is not seen in unsynchronized early S cells (Figure 5B; indicated by black arrow). This pattern suggests pronounced replication in this region due to activation of a previously unseen origin that localizes near mapped ORC1/CDC6 binding sites; intriguingly, this S/G2 peak may correspond with a peak in unsynchronized cells that was only detectable in late S cells (Figure 4). Thus, this may be a weak, late-acting origin that assumes greater importance when replication from the main, centromeric origins of replication (normally responsible for chromosome 1 duplication) is perturbed. These data suggest that ORC1/CDC6 binding sites could be dormant origins ready to be activated in order to ensure the timely replication of the entire chromosome during S phase.

## DISCUSSION

In the present study, we used SMARD methodology for the analysis of DNA replication in *T. brucei* cells. The direct labeling of replicating DNA molecules enabled the visualization and mapping of DNA replication in two life cycle stages of *T. brucei*. We find that the *T. brucei* bloodstream DNA replication rate is 15% faster than procyclic DNA replication rate, and these rates appear to be similar to rates seen in yeast (2 kb/min) (30) and mammalian cells (2–3 kb/min) (31,32). Analysis of a 347 kb region in the middle of chromosome 1 has revealed previously unseen complexity in *T. brucei* replication dynamics. SMARD analysis demonstrated pronounced replication termination within this region, which can only be explained by replication fork progression from both sides, thereby revealing previously unmapped origins. Replication from right to left in the 347-kb region is consistent with two predicted origins located 3′ and centered on the centromere in chromosome 1 (20). Replication from left to right reveals origin(s) 5′ of the 347 kb region that were not seen by MFAseq analysis (20). There may be a number of explanations for this. The leftmost ∼0.2 Mb of chromosome 1 is subtelomeric, where extensive sequence repetition makes read mapping problematic, meaning that a discrete origin may have gone undetected. ORC1/CDC6 mapping was determined by Chromatin Immunoprecipitation (ChIP)-chip analysis, and revealed extensive binding of this initiator to all subtelomeres, with at least five ORC1/CDC6 sites to the left of the 347 kb region of chromosome 1. It is therefore possible that some or all of these sites are potential origins. Not all of these origins need necessarily fire: considering the *T. brucei* procyclic form replication rate of 3.7 kb/min, theoretically only two bi-directional origins of replication may be sufficient to completely replicate all ∼1.1 Mb of chromosome 1 during S-phase, which has been estimated to last for ∼90 mins (27). If a different origin was selected in each cell from the larger pool of ORC1/CDC6 binding sites, this may explain why these origins were not detected by MFAseq analysis (20), since a discrete site of initiation would be lost in the population. Indeed, this might be consistent with the variability in termination site location within the 347 kb region. Moreover, in this regard *T. brucei* might be similar to mammalian cells, where random activation of one origin inhibits activation of other origins in close proximity (33). The predicted leftmost origin may not be in the subtelomere, however, but may localize to the telomere itself, since *T. brucei* ORC1/CDC6 has been shown to bind telomere repeats (16). The differences in termination location in the 347 kb region may be due to stalled or delayed movement of replication forks emerging from the centromeric region caused by head-on encounters with transcription machinery (34,35), since most of the multigenic transcription moves in the opposite direction of this fork (20,36). Alternatively, the variation in termination sites may be due to altered rates of replication progression through the variable subtelomere. Importantly, and irrespective of the precise locations of the putative telomere-proximal origin(s) in chromosome 1, our findings imply that such origin activity may hold true for all chromosomes, meaning that there are more replication origins in *T. brucei* than previously estimated and not limited to the well-conserved chromosome cores (20).

**Figure 6.**
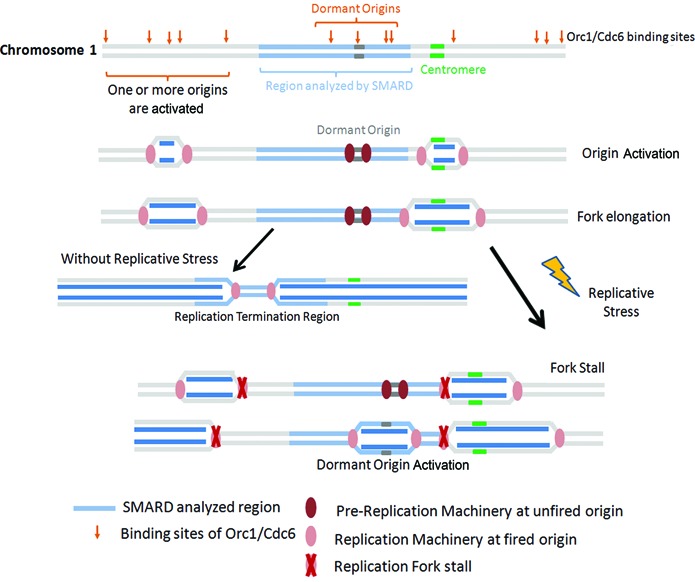
Proposed model for dormant origin activation. At least two origins of replication are fired and are able to complete chromosome 1 replication under normal, unstressed conditions (left): the origin near the centromere and one origin upstream of the SMARD analyzed region (blue region on chromosome 1). However, in a replicative stress condition (right), the forks coming from 5′ and 3′ to the chromosome 1 central region stall, leading to dormant origin activation.

In addition to the description of new origins in unperturbed *T. brucei* cells, we provide the first evidence for dormant origins in this parasite. As shown in Figure 5B, we were able to identify a peak of replication initiation after HU treatment that was not seen in previous MFAseq of untreated cells. The location of this peak appears to overlap with 1–2 mapped ORC1/CDC6 binding sites (20), suggesting that either of these may be the site of the activated origin, which would be consistent with origins that are activated from existing pre-RC complexes when a DNA replication fork is stalled or collapsed, as seen in other eukaryotes (37,38). We were able to detect this putative dormant origin by treating procyclic form *T. brucei* cells with 0.2 mM HU, where nuclear replication is not arrested, but slowed (22). The qPCR data appear to suggest that the effect of the HU treatment is similar that described in other eukaryotes (29,39), with reduction in the dNTP pool causing slowing of the progress of the centromere-derived replication fork (Figure 6, left). Whether this is because the replicative DNA polymerases are impaired in function, or because repair DNA polymerases are predominantly affected (22), is unclear. Nonetheless, the treatment results in a dormant origin, located at the middle of chromosome 1, being activated, most likely in order to ensure the completion of chromosome replication (Figure 6). The presence of dormant origins in *T. brucei* has been previously suggested (20), but has not been demonstrated. Our findings indicate that *T. brucei* has mechanisms to deal with challenges to replication, specifically, the activation of dormant origins. Other mechanisms may also exist, including bypass of replication challenges by translesion DNA synthesis, as recently described (40). Moreover, how many dormant origins there are in *T. brucei*, and where they are located, will require whole genome analysis. Equally, whether the same fail-safe mechanisms for replication rescue are present in related kinetoplastid parasites is currently unknown.

In summary, our data support the existence of more replication origins in *T. brucei* than previously estimated. We find that the replication of *T. brucei* chromosome 1 under normal conditions involves replication forks initiating from origins very close to the centromere region, as already described (20), and from additional origins in the 5′ region of chromosome 1. Activation of one origin in each region would be sufficient to replicate the entire chromosome during unperturbed S-phase (Figure 6, left). However, under replicative stress dormant origin (s) is (are) activated (Figure 6, right). Our findings provide new insights into *T. brucei* genomic duplication, opening new questions concerning the mechanisms and regulation of its DNA replication.

## SUPPLEMENTARY DATA

Supplementary Data are available at NAR Online.

SUPPLEMENTARY DATA
