# Supplementary material for: Single molecule analysis of Trypanosoma brucei DNA replication dynamics
Source: Nucleic Acids Res. 2015 Feb 17;43(5):2655–65. doi: 10.1093/nar/gku1389 (PMC4357695; doi:10.1093/nar/gku1389)
Supplement: SUPPLEMENTARY DATA [file supp_43_5_2655__index.html]

Single molecule analysis of Trypanosoma brucei DNA replication dynamics — SUPPLEMENTARY DATA 

# Single molecule analysis of *Trypanosoma brucei* DNA replication dynamics

## SUPPLEMENTARY DATA

**Files in this Data Supplement:**

- Figure S1
