# Supplementary material for: Single molecule analysis of Trypanosoma brucei DNA replication dynamics
Source: Nucleic Acids Res. 2015 Feb 17;43(5):2655–65. doi: 10.1093/nar/gku1389 (PMC4357695; doi:10.1093/nar/gku1389)
Supplement: SUPPLEMENTARY DATA [file supp_gku1389_nar-03137-f-2014-File009.pdf]

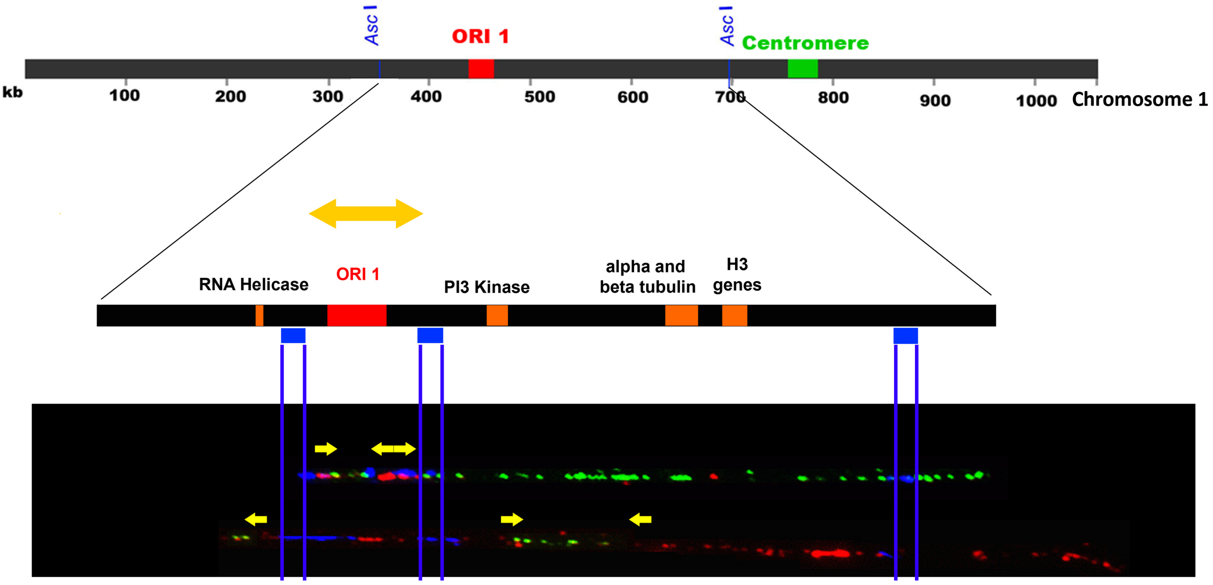

Figure S1: Origin of replication within the 347 kb fragment of chromosome 1. An origin of replication, designated ORI 1 (red box in the chromosome 1), is seen between two of the three FISH probes used. The yellow arrows indicate the direction of replication fork progression.
